# QCloud: A cloud-based quality control system for mass spectrometry-based proteomics laboratories

**DOI:** 10.1371/journal.pone.0189209

**Published:** 2018-01-11

**Authors:** Cristina Chiva, Roger Olivella, Eva Borràs, Guadalupe Espadas, Olga Pastor, Amanda Solé, Eduard Sabidó

**Affiliations:** 1 Proteomics Unit, Centre de Regulació Genòmica (CRG), Barcelona Institute of Science and Technology (BIST), Barcelona, Barcelona; 2 Universitat Pompeu Fabra (UPF), Barcelona, Barcelona; Swiss Institute of Bioinformatics, SWITZERLAND

## Abstract

The increasing number of biomedical and translational applications in mass spectrometry-based proteomics poses new analytical challenges and raises the need for automated quality control systems. Despite previous efforts to set standard file formats, data processing workflows and key evaluation parameters for quality control, automated quality control systems are not yet widespread among proteomics laboratories, which limits the acquisition of high-quality results, inter-laboratory comparisons and the assessment of variability of instrumental platforms. Here we present QCloud, a cloud-based system to support proteomics laboratories in daily quality assessment using a user-friendly interface, easy setup, automated data processing and archiving, and unbiased instrument evaluation. QCloud supports the most common targeted and untargeted proteomics workflows, it accepts data formats from different vendors and it enables the annotation of acquired data and reporting incidences. A complete version of the QCloud system has successfully been developed and it is now open to the proteomics community (http://qcloud.crg.eu). QCloud system is an open source project, publicly available under a Creative Commons License Attribution-ShareAlike 4.0.

## Introduction

Proteomics technologies have matured into a panoply of reliable methods for measuring with high sensitivity thousands of peptides in multiple biological samples. Persistent methodological developments have enabled new large-scope applications for clinical and translational proteomics research [1–3] in which hundreds of samples are prepared and analysed by mass spectrometry. These applications have increased the analytical challenges of proteomics experiments, and generated the need to implement systems to control and assure the quality of each of the steps involved in the proteomics workflow, including sample preparation, chromatographic peptide separation, mass spectrometric acquisition, and data analysis [4–7].

Several community efforts aimed to define common standards [8–11]and to establish quality control and quality assurance procedures have emerged to reduce the variance of proteomics results and to increase their robustness and reproducibility [12,13]. The initiatives arisen within organizations such as the ABRF, HuPO, NCI, and ProteoRed [14–18] reflect the importance of standardization and quality systems. These quality systems are not only required by regulatory agencies to accept proteomics data within clinical contexts [19], but also to increase the analytical throughput in proteomics laboratories by shifting from a reactive action paradigm when problems occur, towards proactive actions induced by an early detection of potential future nonconformities.

Particularly active has been the development of quality control tools to monitor the performance of LC-MS systems in proteomics analyses [20–23]. An extensive set of quality metrics was initially introduced by NIST to assess data quality in proteomics experiments, and since the pioneering MSQC software was released [24], a succession of software packages and metrics have emerged to automate and facilitate this task.

Quality control systems usually benefit from limited human intervention to reduce subjective evaluations and inconsistencies, and to promote a systematic longitudinal evaluation of the system performance. Therefore, proteomics quality control software has focused on automation capabilities, effortless data collection, objective evaluation, and implementation of user-friendly interfaces to overcome what otherwise would be a tedious and labour-intensive task. In this regard, soon after the release of MSQC, another software package, QuaMeter, was released that made use of existing proteomics software tools to get over some initial limitations of its predecessor in an effort to improve its usability and dissemination among proteomics laboratories. Later, the release of SIMPATIQCO [22], Metriculator [21], and others [25], introduced web-based interfaces, interactive plots and comparison capabilities to assist instrument operators in monitoring quality control metric. Other tools, such as OpenMS [26], iMonDB [27] and AutoQC [23], have also contributed to automate the extraction of quality metrics from raw files and, thus, to generate automatic pipelines for quality control. In parallel, methods for evaluating multivariate quality control metrics have emerged [28–30], and tools from the statistical process control framework have been introduced to evaluate instrument performance and to improve the quality of the process [31], such as SproCoP [32] and MsstatsQC [33]. Two thorough reviews of the computational tools available for quality control in LC-MS proteomics experiments have been recently published [34,35].

Despite all previous work to set standard file formats, data processing workflows, and evaluation of key performance parameters, automated quality control systems are not yet widespread among proteomics laboratories, as the field is resilient to their implementation. There are several reasons for this situation such as the difficulty of deploying existing quality control software made by others, license or technical limitations imposed by certain tools (e.g., limited to a particular vendor), the need of trained operators to make the quality control system work, and the lack of modern user-friendly interfaces to facilitate the revision of the quality metrics, and the management of annotations and nonconformities.

Many of the shortcomings of current proteomics quality control software packages end up in laboratories doing intermittent quality control assessment. The lack of a systematic monitoring of instrument performance limits the acquisition of high-quality results, as well as the assessment of the instrument technical variability, and potential inter-laboratory comparisons.

Several initiatives have benefited from community efforts to assess the reproducibility and repeatability of longitudinal proteomics experiments in multiple laboratories [36–38]. However, these studies are often limited in time and in the number of participating laboratories, and they do not benefit from common tools that in addition to quality assessment, provide an effortless and quick evaluation of an instrument performance (*Instrument QC*), and enable instrument comparison within the same laboratory (*Intra-laboratory QC*), and among laboratories (*Community QC*).

Here we present QCloud, a cloud-based quality control system meant to support proteomics laboratories in longitudinal instrument quality control assessment using a user-friendly interface, easy setup, automated data processing that includes database searching capabilities, and unbiased instrument evaluation. The QCloud system offers an automated ready-to-use quality control system to the proteomics operator, who is thus released from software deployment, and method selection to evaluate multivariate metrics related to instrument performance. By reducing the workload associated to quality control, the QCloud system aims to spread the adoption of an automated quality control system to multiple laboratories thus facilitating early detection of instrumental problems and enabling quality control features that align with on-going efforts and agreed standards.

## Materials and methods

### Quality control samples

QC1 samples tested in Thermo and Sciex instruments (LTQ-Orbitrap Velos Pro, LTQ-Orbitrap XL, LTQ-q-Orbitrap Fusion Lumos; and QqQ 5500 QTRAP) corresponded to 1 vial of 500 pmol of commercially available “Trypsin-digested BSA MS Standard (CAM modified)” from New England Biolabs with part number P8108S. The 500 pmol of dried digested bovine serum albumin (BSA) were dissolved with 500 μL of 0.1% formic acid in water and then 15 μL were diluted with 285 μL of 0.1% formic acid in water to 50 fmol/μL. A total of 0.5 μL were injected in each analysis, which corresponded to 25 fmol of BSA.

QC2 samples tested in Thermo and Sciex instruments corresponded to Pierce HeLa protein digest standard from Thermo Fisher Scientific (Part number: 88329). The commercial product is a vial of 20 μg dried digested HeLa extract which are dissolved in 200 μL of 0.1% formic acid in water to a final concentration of 100 ng/μL. A total amount of 1 μL (100ng) is injected per analysis.

All quality control samples used in this work were commercially available.

### QC methods

Quality control samples were analyzed in a LTQ-Orbitrap XL, a LTQ-Orbitrap Velos Pro and a LTQ-Orbitrap Fusion Lumos (Thermo Fisher Scientific) coupled to a Proxeon 1000 nano-LC (Proxeon), and a QqQ 5500 QTRAP (Sciex) coupled to an Eksigent nano-LC Ultra 1D. The liquid chromatography systems were equipped with a C18 reversed-phase chromatography column with column lengths varying from 12 to 50 cm.

Chromatographic gradients for the analysis of quality control samples started at 97% buffer A and 3% buffer B with a flow rate of 250–300 nl/min, and gradually increased to 93% buffer A and 7% buffer B in 1 min, and to 65% buffer A and 35% buffer B in 10–20 min (QC1) and 90–120 min (QC2). After each analysis, the column was washed for 15 min with 10% buffer A and 90% buffer B. Buffer A: 0.1% formic acid in water. Buffer B: 0.1% formic acid in acetonitrile.

Mass spectrometers were operated in positive ionization mode using either a selected reaction monitoring acquisition (20 ms of dwell time, 5500 QTRAP), or data-dependent acquisition (Orbitrap-based instruments) in which each survey scan was followed by the fragmentation of the top *n* most intense multiple charged ions via collision-induced dissociation (CID). Dynamic exclusion, resolution, injection and accumulation times, and the number of fragmented precursor ions were tuned in each LC-MS system and type of QC sample to obtain a cycle time that results in 8–10 data points per chromatographic peak.

All data were acquired with the software Xcalibur v2.2, and Analyst v1.6.

## Results and discussion

### Overview of the QCloud system

QCloud is a cloud-based quality control system that establishes a seamless quality control pipeline and, thus, eliminates those barriers that usually prevent the adoption of quality control tools as an integral part of mass spectrometry proteomics workflows.

**Fig 1:**
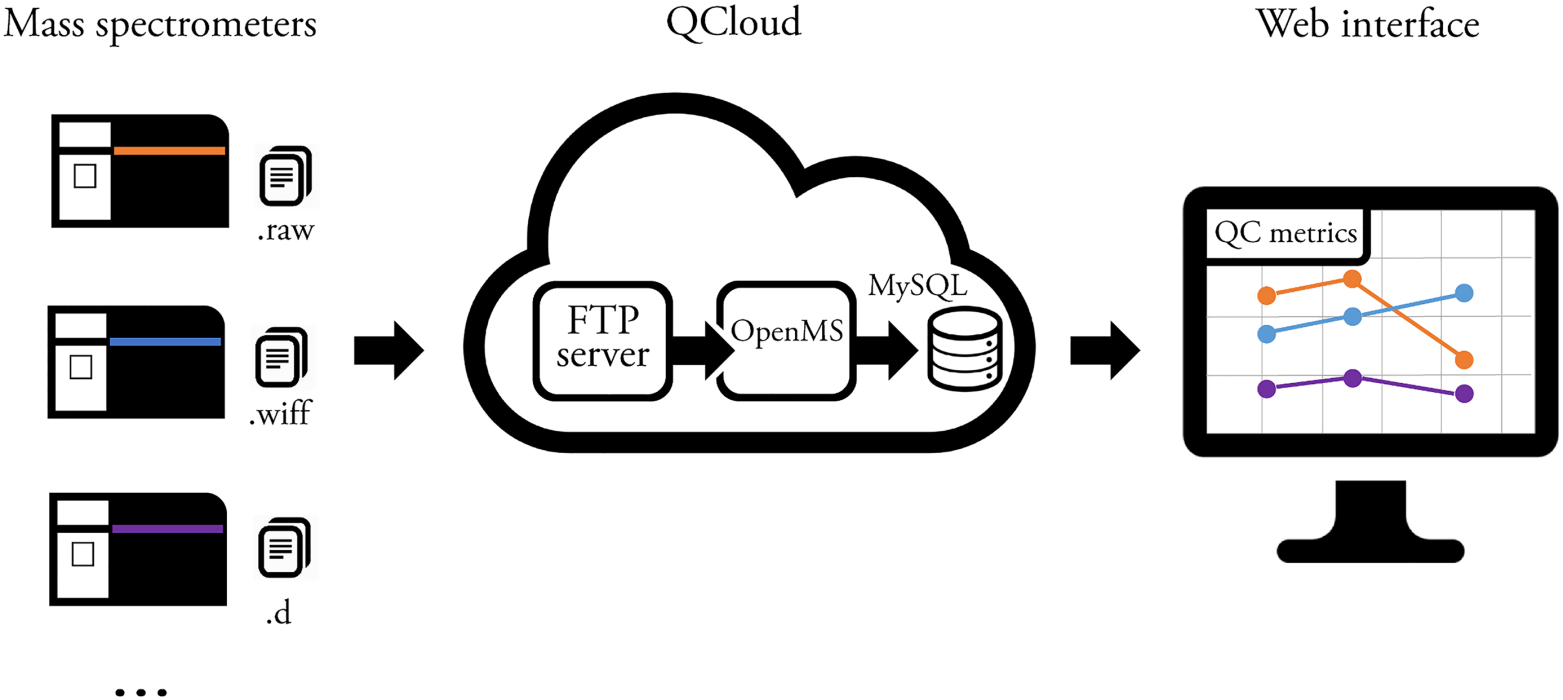
QCloud overview. Overview of the QCloud system structure consisting in i) a thin client in the mass spectrometer acquisition computer, ii) the cloud-based processing infrastructure, and iii) the web user interface.

The QCloud system consists in i) a thin client installed in the mass spectrometer acquisition computer, ii) a cloud-based processing infrastructure, and iii) a web user interface (Fig 1) that automate the complete quality control workflow by performing automatic data collection from the instrument, data processing, unbiased instrument evaluation, metrics display, and data self-archiving. Compared to existing proteomics quality control systems, QCloud greatly facilitates the initial system setup to mass spectrometer operators and significantly reduces the time and efforts required by a proteomics laboratory to adopt a quality control system for continuous performance assessment of mass spectrometry-based proteomics experiments.

QCloud relies on previous community efforts and the system development has been based on the Java programming language, msconvert [39], the OpenMS infrastructure [26], the qcML data format [11], and a LAMP webserver. QCloud is an id-based quality control system and it currently supports different proteomics workflows, including shotgun discovery proteomics, peptide MS1 quantitation, parallel reaction monitoring, and selected reaction monitoring.

The QCloud system has been designed to assess the performance of the LC-MS systems in any laboratory and it can adopt data files from different mass spectrometer vendors. Currently, the QCloud system has been tested on Thermo and Sciex instruments, specifically the LTQ-Orbitrap XL, LTQ-Orbitrap Velos Pro, LTQ-q-Orbitrap Fusion Lumos, Q-Exactive series, as well as on the QqQ QTRAP 5500.

A complete version of the QCloud system has successfully been developed and it is now open to the proteomics community (http://qcloud.crg.eu).

### Quality control samples

The QCloud supports two types of quality control samples, QC1 and QC2, according to the nomenclature established by Pichler et al. In 2012 [22]. The QC1 sample corresponds to a low complexity quality control sample that is analysed several times per day in order to establish the performance of the instrument before and after running each real sample. In contrast, the QC2 sample is a high complexity sample that mimics real samples analysed in a proteomics laboratory, and is meant to be injected 1–5 times per week as a sample to test system suitability.

Currently the QCloud system supports bovine serum albumin as QC1 sample, and the HeLa cellular digested proteome as QC2 sample. Supported quality control samples were selected based on their commercial availability as ready-to-inject samples to reduce sample manipulation within the laboratory and to facilitate sample preparation, accessibility and batch tracking. The use of pre-defined, ready-to-use, and easily accessible quality control samples does not only facilitate workflow automation, but also instrument comparison within the same laboratory (*Intra-laboratory QC*), and among laboratories (*Community QC*).

Although a yeast QC reference material has been defined by NIST [40], this QC sample is not currently supported by QCloud due to its limited availability and non-sustained supply. However, the QCloud framework allows the easy incorporation of new reference materials as soon as they are defined and made broadly accessible.

### Detailed description of the QCloud system

Quality control samples need to be regularly programmed for analysis by the mass spectrometer operator following the recommended acquisition methods (see “Materials and methods*”* section). QC1 samples are analysed with short methods of 10–20 min with narrow chromatographic peak widths and short cycle times, whereas standard high complexity methods of 90–120 min are recommended for the analysis of QC2 samples. Despite the definition of several recommended methods, parameters might need to be adapted when other types of mass spectrometers, chromatographic systems, solvents and chromatographic columns are used.

**Fig 2:**
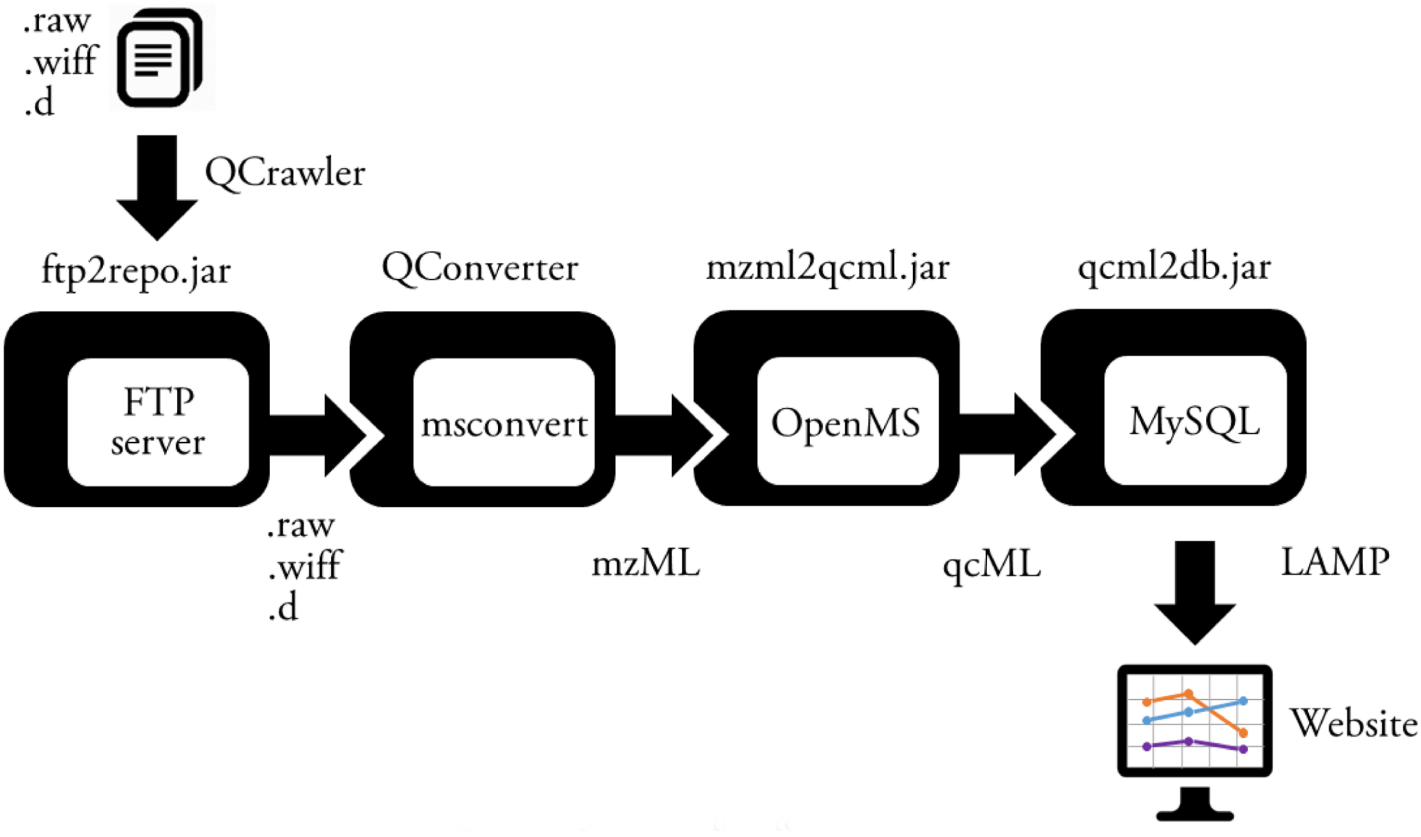
Detailed scheme of the cloud-based processing infrastructure pipeline.

By using a defined file naming system, the created quality control raw files are automatically processed by QCrawler, a thin client (374 KB) that needs to be run in the instrument computer. QCrawler is, therefore, the entry point into the QCloud system. It performs user authentication, and once the user has defined the acquisition folder and the instrument, it automatically locates and uploads the quality control acquisition files and instrument parameters to the QCloud system through a remote FTP server (Fig 2). QCrawler is coded with the.NET v3.0 framework to be retro-compatible with a wide range of versions of the Microsoft Windows™ operating system installed in the acquisition computers.

Quality control sample raw files are then converted to the mzML open standard using the QConverter wrapper of msconvert (v.3.0.9393) in a Microsoft Windows™ virtual machine and the spectra are processed via a Java-based wrapper of OpenMS v2.0 (mzml2qcml.jar). The system adapts to data files from different mass spectrometer vendors. QCloud currently supports both id-free and id-based proteomics workflows, thus including targeted methods such as precursor ion quantitation, parallel reaction monitoring, and selected reaction monitoring, as well as untargeted workflows such as shotgun discovery proteomics. On shotgun proteomics samples, QCloud relies on a database search with the OMSSA search engine and the FeatureFinder module in OpenMS, whereas in targeted proteomics the fragment ion chromatograms are extracted for a selection of bovine serum albumin peptides (see detailed workflows at https://github.com/rolivella/QCloud).

**Fig 3:**
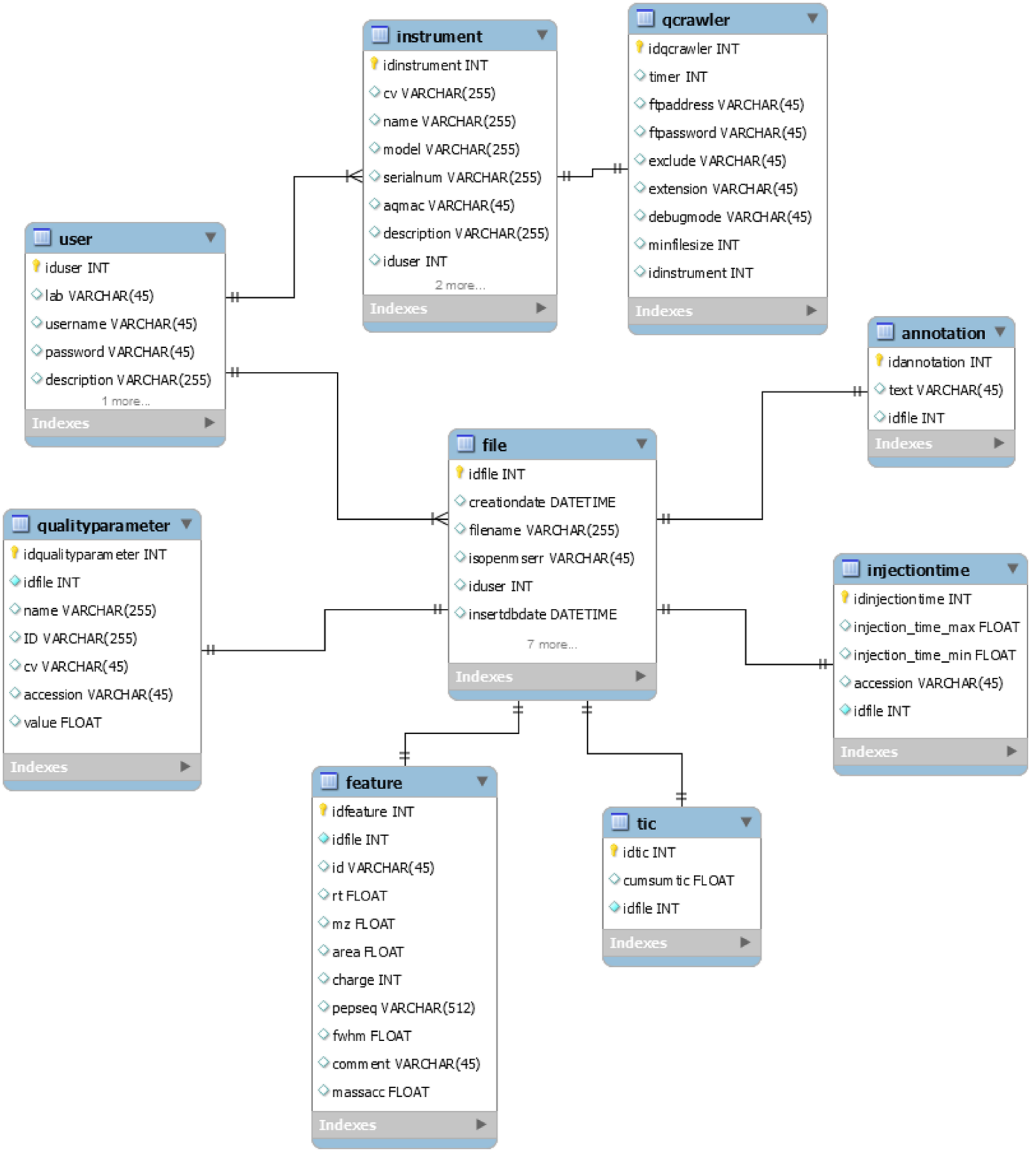
Table scheme and relationship of the persistent layer.

**Fig 4:**
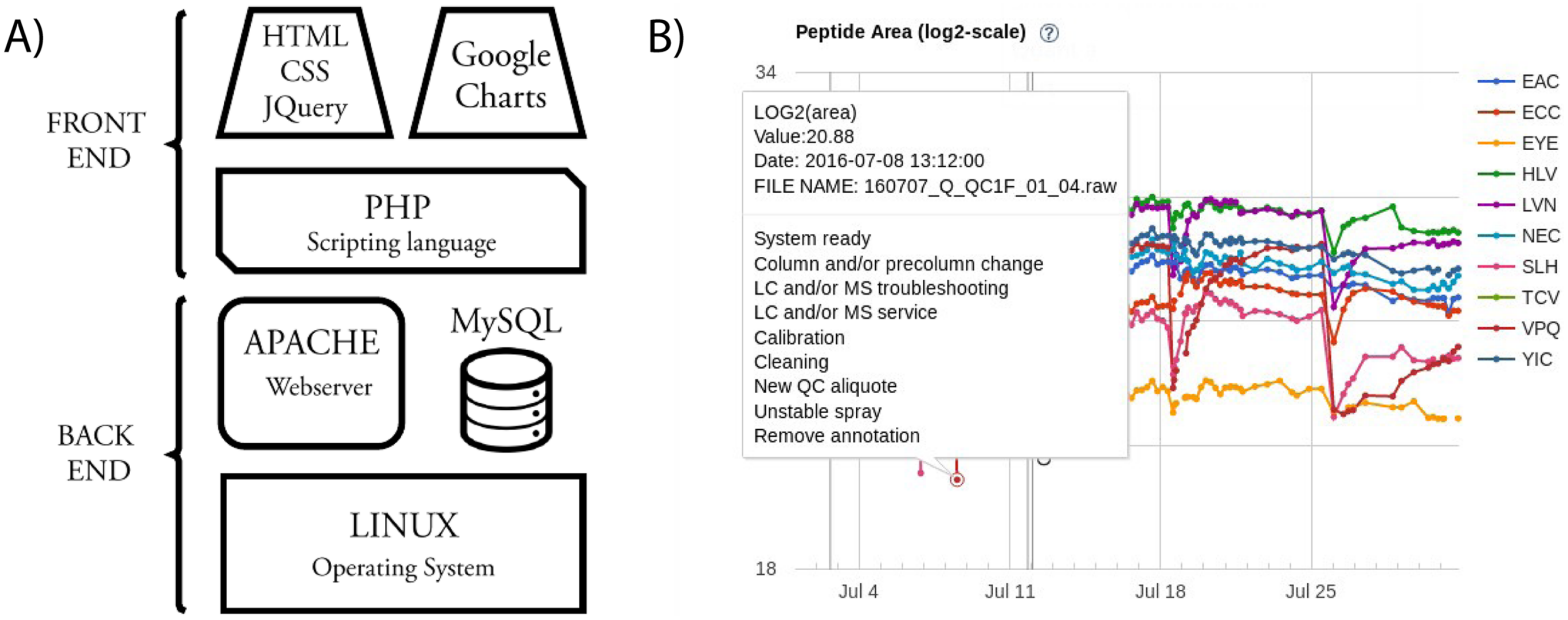
Web server front-end. A) Schematic architecture of the server back and front-end. B) Example of quality control data point annotations with controlled vocabulary in the QCloud system in a profile plot of log2(Area) of multiple selected peptides (see Table 2).

A set of quality control parameters is extracted from the qcML, idXML and featureXML files (see Tables 1 and 2) and they are stored in the persistence layer by a MySQL wrapper (qcml2db.jar, Fig 3). Quality control data are presented to the user in a web server front-end with a responsive layout based on jQuery, CSS bootstrap, and Google Charts (Fig 4A). The user interface is interactive and it enables user annotations with controlled vocabulary to report incidences and annotate the acquired data, potential problems with nonconformities, and operator interventions (Fig 4B).

**Table 1:** List of quality control parameters currently extracted by QCloud.

Parameter name	CV HuPO-PSI	NIST	Description
Peak Area			Areas of the features corresponding to the list of selected peptides within a mz (+/- 5 ppm) and RT (+/-240 s) tolerance window
Mass Accuracy	QC:0000038		1E6 x (observed_mz-theoretical_mz)/theoretical_mz where the observed_mz is extracted from the featureXML
Retention Time Drift			Peptide retention time difference between the current QC sample and the previous one
Median Injection Time MS1	Median MS:1000927 (MS1-only)	MS1-1 (similar)	Median ion injection time of all MS1 scans.
Median Injection Time MS2	Median MS:1000927 (MS2-only)	MS2-1 (similar)	Median ion injection time of all MS2 scans.
Chromatographic Resolution			(RT pep1 –RT pep2) / (FWHM(pep1) + FWHM(pep2))*
Peak Capacity			(max(RT)–min(RT)) / (average(FWHM))
Total Ion Current	QC:0000048	MS1-2B (similar)	Sum of all TIC per RT extracted from the qcML
MS1 Spectra Count	QC:0000006	DS-2A (similar)	
MS2 Spectra Count	QC:0000007	DS-2B (similar)	
Chromatogram Count	QC:0000008		
TIC Slump	QC:0000023		
Total Number of Missed Cleavages	QC:0000037		
Total Number of Identified Proteins	QC:0000032		
Total Number of Uniquely Identified Proteins	QC:0000033		
Total Number of PSMs	QC:0000029	P-2A	
Total Number of Identified Peptides	QC:0000030	P-2B	
Total Number of Uniquely Identified Peptides	QC:0000031	P-2C	
Mean Delta ppm	QC:0000040		
Median Delta ppm	QC:0000041	MS1-5C	
Id Ratio	QC:0000035		
MS Quantification Results Details	QC:0000045		
Number of Features	QC:0000046		

* This parameter is calculated with two peptide pairs: the first pair is pep1 "SLADELALVDVLEDK" and pep2 "RFPGYDSESK", and the second pair is pep1 "FEELNMDLFR" and pep2 "LAVDEEENADNNTK".

**Table 2:** List of monitored peptides for QC1 and QC2 samples.

QC1	QC2
LVNELTEFAK	YAEAVTR
HLVDEPQNLIK	TPAQFDADELR
VPQVSTPTLVEVSR	STLTDSLVC(CAM)K
EAC(CAM)FAVEGPK	SLADELALVDVLEDK
EYEATLEEC(CAM)C(CAM)AK	NPDDITNEEYGEFYK
EC(CAM)C(CAM)HGDLLEC(CAM)ADDR	LGDLYEEEMR
SLHTLFGDELC(CAM)K	LAVDEEENADNNTK
TC(CAM)VADESHAGC(CAM)EK	FEELNMDLFR
YIC(CAM)DNQDTISSK	EAALSTALSEK
NEC(CAM)FLSHK	DDVAQTDLLQIDPNFGSK
	RFPGYDSESK
	EVSTYIK
	EATTEFSVDAR
	FAFQAEVNR
	EQFLDGDGWTSR

**Fig 5:**
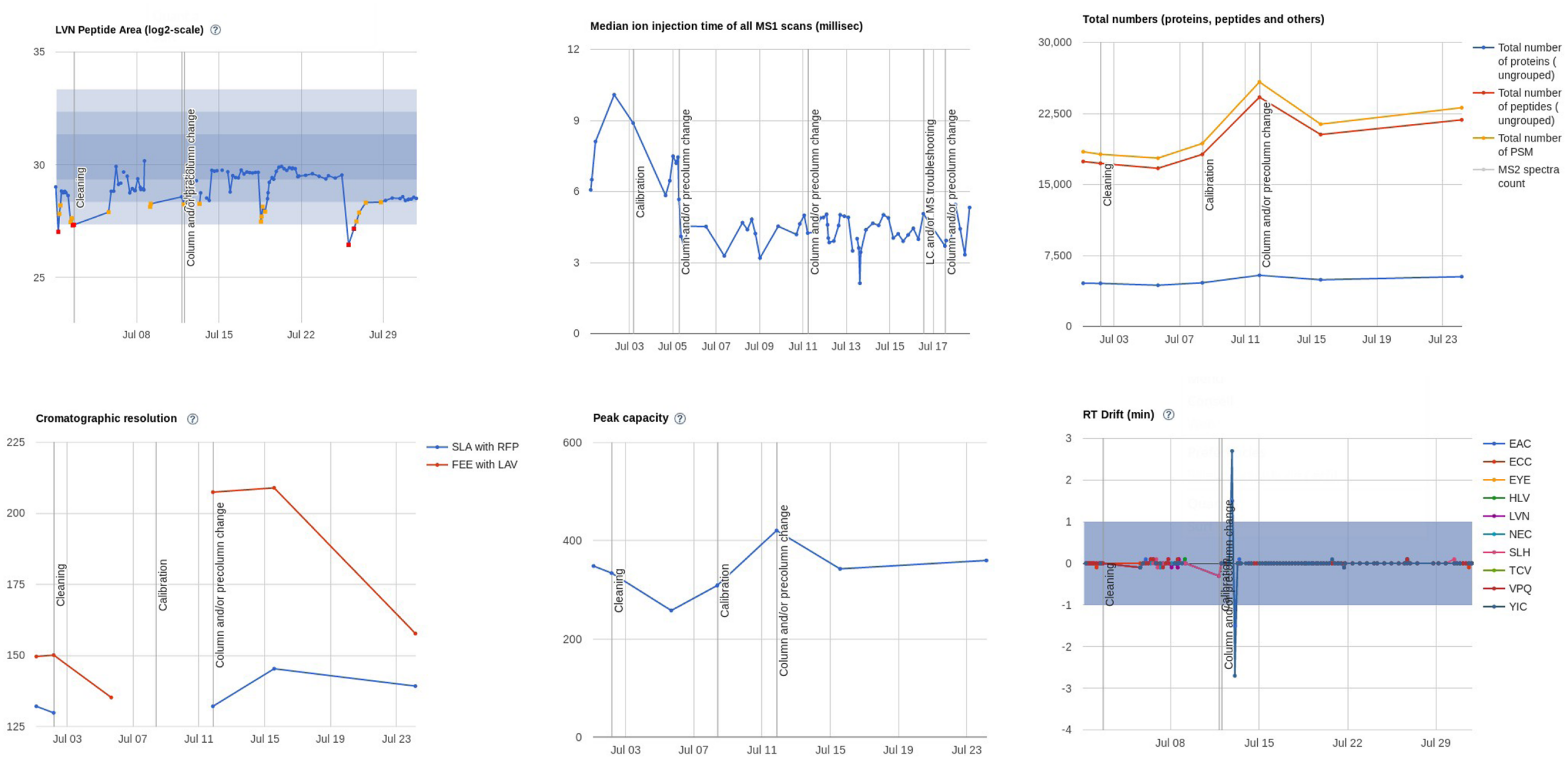
Quality control charts. A sample of several quality control charts displayed in the web interface by the QCloud system, including peptide areas, injection time, total numbers of proteins, peptides and PSM, chromatographic resolution, peak capacity, and retention time drift. Plotted parameters are defined in Table 1.

Quality control parameters are subjected to statistical assessment based on their comparison with a high-performance period defined by the user, which is used to set the acceptance thresholds. QCloud classifies each data point in three categories: a) *conformities* (green) when the parameter lays within two standard deviations around its mean, b) *warnings* (yellow) when the parameter is between two and three standard deviations around its mean, and *nonconformities* (red) when the assessed value is beyond three standard deviations from its mean. This setup enables automated non-subjective instrument performance evaluation and the definition of the three categories directly translates into specific actions—i.e. continue acquiring, do a preventive action, or stop the instrument, respectively (Fig 5).

The code for the QCloud system is publicly available at https://github.com/rolivella/QCloud under a Creative Commons License Attribution-ShareAlike 4.0 and it accepts contributions from the community.

### Benefits and limitations of the QCloud system

**Fig 6:**
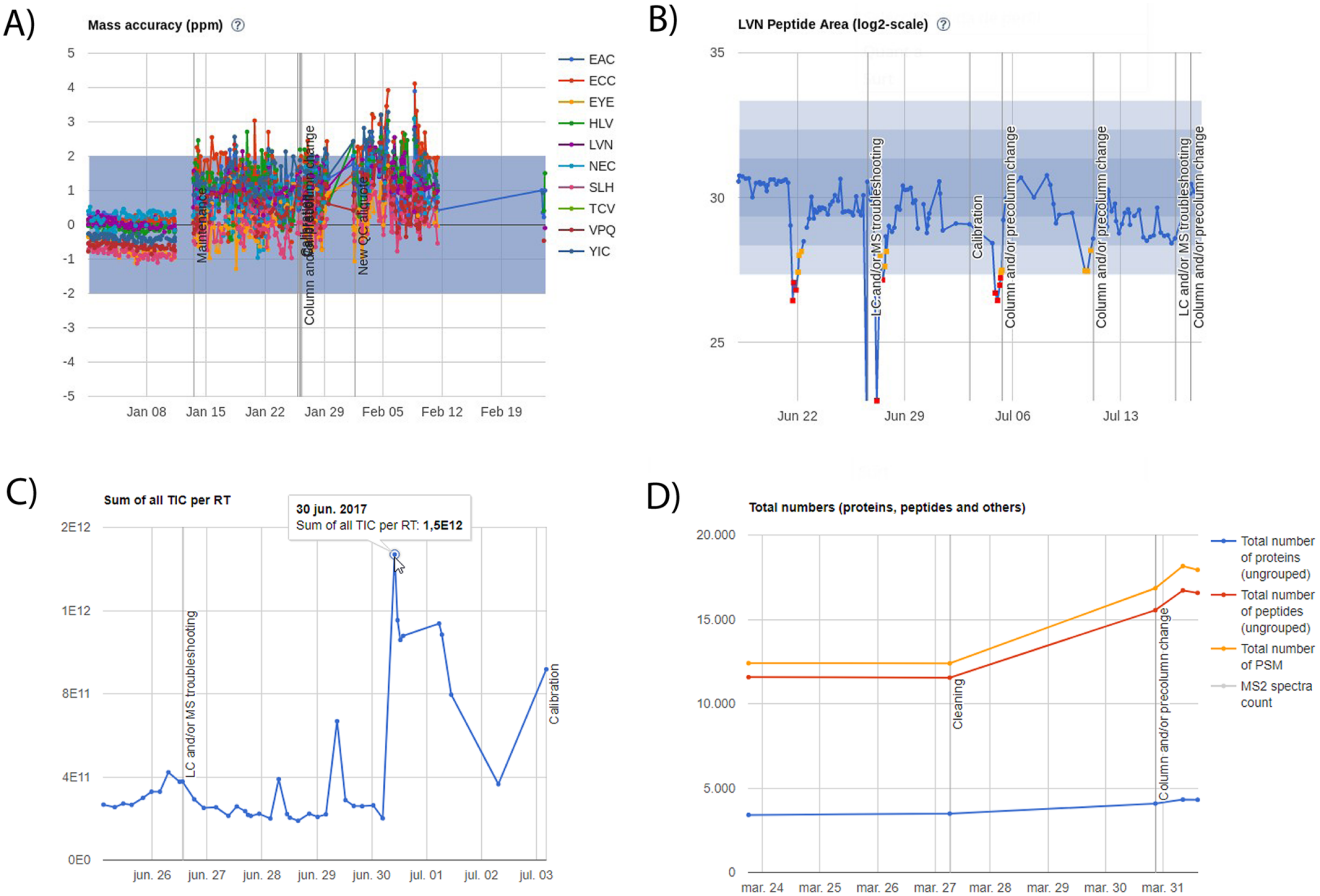
Troubleshooting with QCloud. A) Example of slight mass calibration problems identified using QC1 controls after an instrument maintenance procedure; B) Sudden losses of performance classified as non-conformities QC1 samples (red dots) that triggered maintenance interventions annotated as vertical lines; C) Example of sample carry over detected in the TIC plot of one QC1 sample acquired after a problematic sample; D) Increase in the total number of PSMs, peptides, and proteins from a QC2 quality control sample after a cleaning routine. All plots correspond to quality control data generated in an Orbitrap Fusion Lumos.

At this point, QCloud has been up and running for more than one year providing quality control services to several proteomics laboratories from different research institutions, including ours. In our laboratory, QCloud performs unattended processing of an average of ten QC1 samples per day and per instrument, and two QC2 samples per week and per instrument, which results in more than 3,000 quality control samples automatically processed per year in each instrument. During this period, QCloud allowed us to pinpoint a variety of incidences, including slight mass calibration problems, sudden loss of performance, sample carry over, and it provided an objective classification of conformities and non-conformities that determined when operator interventions were needed (Fig 6). Early detection of instrument incidences allowed us to perform correction actions at early stages of the proteomics workflow, thus saving time, money and samples, compared to post-acquisition quality control evaluation.

Compared to other available proteomics quality control software, QCloud offers a complete automation of the quality control workflow, covering all the required steps from the raw files to the automatic generation of plots in the web user interface. Therefore, QCloud releases the operator from the need to manually copy, upload, and process any quality control raw file to extract and plot the metrics of interest. By reducing the workload associated to quality control, the system facilitates the adoption of quality control system in proteomics laboratories, as one person can manage the quality control of several instruments while attending other requirements from the laboratory. Moreover, the system offers a single entry-point for objective evaluation of instrument performance to all laboratory operators, thus avoiding discrepancies in quality control assessment.

Currently, QCloud performs the whole quality control process—from sample injection to the publication of the quality control metrics in the website—with less than one hour when a network connection >10 Mbps is available. This includes file upload (1–5 min), file conversion (2–10 min), id-based and/or id-free analysis workflows (5–40 min), plus small synchronization overheads. Overall, a shotgun QC1 BSAs sample analysed with database search takes around 10–15 min to appear in the website, whereas a QC2 HeLa sample takes around 30–45 min. Therefore, QCloud does not offer real-time data analysis for on-the-fly interventions, but the data processing time is fast enough to generate a quality evaluation while the next sample is being acquired.

Finally, it is worth mentioning, that QCloud supports a limited set of quality control metrics, acquisition workflows and samples—mainly bovine serum albumin for QC1 samples, and HeLa cell extracts for QC2 samples. Therefore, compared to other quality control software or workflow generator frameworks, QCloud is a streamlined system that offers little customization to the user. This simplifies software maintenance and it enables the comparison of instrument performance within or among different laboratories.

## Conclusions

Comprehensive quality control systems need to become an integral part of mass spectrometry-based proteomics experiments. This will facilitate objective and systematic longitudinal evaluations of system performance and, thus, advance into robust and reproducible proteomics analyses in the fields of clinical and translational proteomics research.

Substantial community efforts have led to significant advances in data standardization, definition of performance parameters, and development of quality control software and statistical assessment methods. To transform these efforts into a wide adoption of quality control system in proteomics laboratories, advances are required in the cost-benefit curve of academic software towards the development of user-friendly and easy-to-setup systems.

The QCloud quality control system belongs specifically into this niche, as it aligns with on-going community efforts and agreed standards to establish a seamless quality control pipeline. QCloud is a cloud-based longitudinal quality control system for MS-based proteomics that relies on the Java programming language, the OpenMS infrastructure, the qcML data format, and a LAMP web server. The system eliminates the barriers that usually prevent the adoption of quality control tools and offers a ready-to-use system to proteomics laboratories. Indeed, QCloud does not require software deployment, it supports several vendors and acquisition workflows, and it offers a modern user interface that facilitates the revision of the quality parameters, and the annotation of nonconformities.

It has not escaped our notice that cloud-based systems have the potential not only to facilitate instrument comparison within the same laboratory, but also among different laboratories (*Community QC*). The implementation of new community features will thus enable the performance comparison of a given instrument with the average performance of the community, and identify the most common causes of performance problems and downtime. Moreover, cloud-based quality control tools will progressively support advanced multivariate statistical methods, automated notifications, and additional proteomics workflows such as sample preparation. Finally, we envisage the implementation of smart actions to control the acquisition flow according to quality parameters (*e*. *g*., stop acquisition queue or program washing routines), making use of the advanced API interfaces available in modern mass spectrometers.

In conclusion, cloud-based ready-to-use tools, such as QCloud, will accelerate the adoption of quality control systems for continuous quality assessment in proteomics laboratories and provide an effortless and quick evaluation of instrument performance.
